# Rapid local adaptation linked with phenotypic plasticity

**DOI:** 10.1002/evl3.176

**Published:** 2020-05-27

**Authors:** Syuan‐Jyun Sun, Andrew M. Catherall, Sonia Pascoal, Benjamin J. M. Jarrett, Sara E. Miller, Michael J. Sheehan, Rebecca M. Kilner

**Affiliations:** ^1^ Department of Zoology University of Cambridge Cambridge CB2 3EJ United Kingdom; ^2^ Department of Entomology Michigan State University East Lansing Michigan 48824; ^3^ Department of Neurobiology and Behavior Cornell University Ithaca New York 14853

**Keywords:** Burying beetles, interspecific competition, local adaptation, niche expansion, *Nicrophorus vespilloides*, phenotypic plasticity, plasticity‐led evolution

## Abstract

Models of “plasticity‐first” evolution are attractive because they explain the rapid evolution of new complex adaptations. Nevertheless, it is unclear whether plasticity can facilitate rapid microevolutionary change between diverging populations. Here, we show how plasticity may have generated adaptive differences in fecundity between neighboring wild populations of burying beetles *Nicrophorus vespilloides*. These populations occupy distinct Cambridgeshire woodlands that are just 2.5 km apart and that probably originated from a common ancestral population about 1000‐4000 years ago. We find that populations are divergently adapted to breed on differently sized carrion. Adaptive differences in clutch size and egg size are associated with divergence at loci connected with oogenesis. The populations differ specifically in the elevation of the reaction norm linking clutch size to carrion size (i.e., genetic accommodation), and in the likelihood that surplus offspring will be lost after hatching. We suggest that these two processes may have facilitated rapid local adaptation on a fine‐grained spatial scale.

**Impact Summary**

Pre‐existing phenotypic plasticity is thought to enable the rapid evolution of new adaptations. However, it is not clear exactly how rapidly new adaptations might arise through this process, nor whether adaptations can be finely matched to local conditions. We addressed this problem by studying neighboring populations of wild burying beetles *Nicrophorus vespilloides* in two English woodlands that are just 2.5 km apart. Each wood is a fragment of the Wild Wood, the ancient forest that covered England until deforestation, about 1000‐4000 years ago.

All the U.K. *Nicrophorus* burying beetle species use small carrion to breed upon and they compete for this scarce resource. We found that these *Nicrophorus* species partition the carrion niche according to their size, with larger beetle species breeding more efficiently on larger corpses. *Nicrophorus vespilloides* is the smallest of these species and it coexists with a different number of *Nicrophorus* burying beetle species in the two woods we studied. In Gamlingay Wood, where the carrion niche is finely subdivided between four species, we found *N. vespilloides* is specialized to breed efficiently on only the smallest carcasses. In Waresley Wood, however, where *N. vespilloides* coexists with a single much larger burying beetle species, it breeds efficiently on carrion that ranges more widely in size. It has expanded into the carrion niche left vacant by the missing two *Nicrophorus* species, which are intermediate in size.

We discovered that the divergence in breeding efficiency is due to divergence in the elevation of the reaction norm linking clutch size to carrion size: Waresley *N. vespilloides* lay more eggs per gram of carrion than Gamlingay *N. vespilloides*. When Waresley *N. vespilloides* breed on smaller carrion, surplus offspring are culled, but on larger carrion Waresley beetles are able to convert the additional resources into larvae. In these ways, phenotypic plasticity has underpinned rapid divergent adaptation in female fecundity, which is finely tuned to local environmental variation.

Understanding the scale and pace of local adaptation is a long‐standing problem in evolutionary biology. It has recently acquired new significance because it can predict how natural populations will respond to man‐made environmental change (Baldwin 1896; Morgan 1896; Robinson and Dukas 1999; West‐Eberhard 2003; Pfennig et al. 2010; Levis and Pfennig 2016). In theory, phenotypic plasticity can potentially both accelerate the pace of evolution and fine‐tune the scale of local adaptation. The extent of phenotypic plasticity in any trait is described by a reaction norm, which relates environmental variation to the phenotype it induces. “Plasticity‐first” evolution can speed up the pace of evolutionary change in complex traits because the shape, slope, and elevation of a reaction norm each have genetic components, upon which selection can act (Baldwin 1896; Morgan 1896; Robinson and Dukas 1999; West‐Eberhard 2003; Pfennig et al. 2010; Levis and Pfennig 2016). New adaptations can evolve through the “genetic assimilation” (sensu Waddington 1953) of traits that were once induced environmentally.

Much of the evidence for “plasticity‐first” evolution derives from interspecific comparisons across million‐year long evolutionary timescales, and it focuses on the origin of discrete and complex novel adaptations (e.g., Gomez‐Mestre and Buchholz 2006; Rohner et al. 2013; Susoy et al. 2015; Corl et al. 2018; Levis et al. 2018). Yet “plasticity‐first” evolution could also facilitate microevolution. Locally adapted traits could result from an evolved change in the gradient or elevation of a reaction norm, for example, they need not involve the complete loss of plasticity altogether through canalization. The evidence for this type of plasticity‐led evolution, however, is scarce.

We tested whether plasticity has facilitated recent, local adaptation in wild populations of burying beetles *Nicrophorus vespilloides*. We focused on populations occupying Gamlingay Wood and Waresley Wood in Cambridgeshire, United Kingdom, which are about 2.5 km apart. Gamlingay Wood and Waresley Wood have existed as distinct woodlands since at least 1086 because they are both recorded in the Domesday Book (a land survey commissioned by William the Conqueror). However, until roughly 3000‐4000 years ago they were almost certainly connected as part of the “Wild Wood,” the ancient forest that once covered England but which was deforested from the Bronze Age onward (Rackham 2000). Furthermore, genetic analyses suggest that *N. vespilloides* populations now occupying Gamlingay and Waresley Woods were recently derived from a single large population of *N. vespilloides* that once occupied the Wild Wood, because they cannot be differentiated at neutral genetic markers (Pascoal and Kilner 2017). We investigated whether *N. vespilloides* has divergently adapted to the contrasting ecological conditions that exist in the relatively new woodland islands of Gamlingay and Waresley Woods.

In the United Kingdom, it is common for up to four *Nicrophorus* species to co‐exist in guilds within each discrete patch of woodland. All *Nicrophorus* spp. breed on small dead vertebrates, such as rodents. If several *Nicrophorus* beetles discover the same carcass, they compete to determine who gets exclusive ownership of the carcass. Contests are typically won by the largest species, or the largest individuals within species (Trumbo 1990). Burying beetles reduce competition between species by partitioning the carrion niche according to their body size. In general, larger *Nicrophorus* species appear to be under selection to breed on larger carcasses (Smith and Heese 1995; Hopwood et al. 2016), forcing smaller beetles to breed on smaller carrion. Accordingly, *N. vespilloides* is more than twice as likely to be found on small carcasses than on large carcasses in continental forests, which are rich in *Nicrophorus* species (Urbański and Baraniak 2015). Burying beetles match the number of eggs they lay to the size of the carcass they obtain (Müller et al. 1990), and facultatively cull any surplus larvae through partial filial cannibalism (Bartlett 1987). The adaptive clutch size and brood size produced by each population therefore depends on the size of the carrion that is routinely available for reproduction.

Here, we show that burying beetle guilds differ between Gamlingay and Waresley Woods, and that this likely changes the size of carrion available for *N. vespilloides* to breed upon in each wood (its “carrion niche,” after Scott 1998). We demonstrate corresponding divergence in levels of fecundity shown by *N. vespilloides* from each population and find associated differences at loci that are likely to underpin this divergence. By characterizing the reaction norm linking fecundity to carrion size for each population, we show how plasticity is linked to such rapid adaptive divergence between neighboring populations.

## Results

### THE *Nicrophorus* GUILD DIFFERS BETWEEN GAMLINGAY AND WARESLEY WOODS

**Figure 1:**
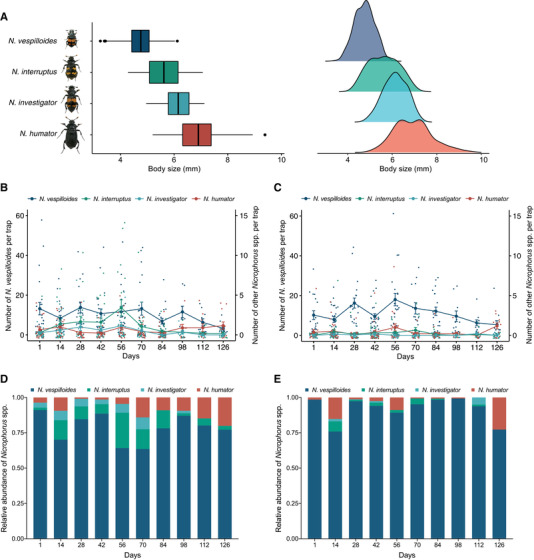
(A) Body size and frequency distribution of field‐caught *Nicrophorus* spp., illustrated with box‐and‐whisker plots (left) and kernel density estimation (right). Points indicate outliers. (B and C) Temporal variation in abundance of *N. vespilloides* (left *y*‐axis) and the other *Nicrophorus* spp. (right *y*‐axis) per trap in (B) Gamlingay and (C) Waresley Woods. The values represent the mean ± SEM of data collected per trap at the same time each year. (D and E) Averaged relative abundance of *Nicrophorus* spp. per trap throughout the field seasons in (D) Gamlingay and (E) Waresley Woods.

Each year, from 2014 to 2017 plus 2019, we set five beetle traps per woodland, at exactly the same five locations within each wood (Fig. S1A), and checked the contents every two to three weeks from June until October, rebaiting the trap each time with fresh compost and a dead mouse. In general, we found that the two woodlands harbored a similar number of *Nicrophorus* beetles: we caught a total of 2219 *Nicrophorus* individuals in Gamlingay Wood over the five‐year sampling period compared with 2096 *Nicrophorus* individuals in Waresley Wood. Although Gamlingay Wood routinely supports four *Nicrophorus* species, Waresley Wood is frequently inhabited by two species, with the other two species much less commonly caught (Fig. 1).

We found that the number of each *Nicrophorus* species per trap varied across the field season (sampling day × species interaction: *χ*² = 190.10, d.f. = 27, *P* < 0.001; Figs. 1B and 1C), and that the number of each *Nicrophorus* species differed between the two woods (species × woodland interaction: *χ*² = 155.67, d.f. = 3, *P* < 0.001; Figs. 1B and 1C). *Nicrophorus vespilloides* was by far the most abundant in each woodland, comprising 81.6% (1811 individuals) of all *Nicrophorus* beetles trapped in Gamlingay Wood and 93.7% (1963 individuals) of those trapped in Waresley Wood. Both sites also contained stable populations of the largest burying beetle species, *N. humator* (Fig. 1A; Tables S1 and S2). Only Gamlingay Wood contained stable populations of intermediate‐sized *N. interruptus* and *N. investigator* in all five years of the study (Fig. 1B‐E; Tables S1 and S2), and in significantly greater abundance in Waresley Wood (Tukey HSD, *z* = 8.68, adj. *P* < 0.001 and *z* = 6.26, adj. *P* < 0.001, respectively). We found that a *N. vespilloides* beetle from Waresley Wood was much less likely than a *N. vespilloides* beetle from Gamlingay Wood to encounter *N. humator*, *N. interruptus*, and *N. investigator* within the same trap (woodland effect: *χ*² = 24.52, d.f. = 1, *P* < 0.001; Fig. 1C; see also Scott 1998).

Similar results were obtained when comparing the relative abundance of each *Nicrophorus* spp. at each trap site, between the two woodlands. There was a seasonal variation for different beetle species in their relative abundance (sampling day × species interaction: *χ*² = 190.60, d.f. = 27, *P* < 0.001), and each *Nicrophorus* spp. differed in their relative abundance between the woodlands (woodland × species interaction: *χ*² = 155.70, d.f. = 3, *P* < 0.001). These results suggest that the relative abundance of *Nicrophorus* spp. varies over time within each woodland, but that overall there are stable, persistent differences between the woodlands in the relative abundance of the different burying beetle species (woodland × species interaction: *χ*² = 5.08, d.f. = 9, *P* = 0.827). There were temporal and spatial variations in the absolute number of *Nicrophorus* species too (see Fig. S2). *Nicrophorus interruptus* and *N. investigator* each comprised a greater proportion of all beetles caught in Gamlingay than Waresley Wood (Tukey HSD, *z* = 9.55, adj. *P* < 0.001 and *z* = 6.40, adj. *P* < 0.001, respectively; Figs. 1D and 1E).

Using different approaches, these analyses consistently show that the *Nicrophorus* guild is significantly different between the two woodlands (PERMANOVA [permutational multivariate ANOVA] test *F* = 5.45, *P* < 0.001; Fig. S1B). Together, they suggest *N. vespilloides* from Waresley Wood is less likely to face competition for carrion from larger congenerics than *N. vespilloides* from Gamlingay Wood.

### NO EVIDENCE FOR A DIFFERENCE IN THE SMALL MAMMAL POPULATION BETWEEN GAMLINGAY AND WARESLEY WOODS

We sampled the small mammal population in Gamlingay and Waresley Woods to estimate the abundance and type of carrion that might be available to the burying beetles to breed upon (see Methods). In Gamlingay, 32 animals were caught across 50 trap sessions (23 new catches and nine recaptures); in Waresley 41 animals were caught across 50 trap sessions (30 new catches and 11 recaptures). Across both woods, bank voles (*Myodes glareolus*; range: 15‐40 g) and wood mice (*Apodemus sylvaticus*; range: 13‐27 g) were the dominant species, constituting 53% and 43% of all trapped mammals, respectively. There was no difference in the mean body mass of small mammals sampled between the two sites (*χ*² = 0.19, d.f. = 1, *P* = 0.661; Fig. S3).

### THE *N. vespilloides* “CARRION NICHE” DIFFERS BETWEEN GAMLINGAY AND WARESLEY WOODS

We conclude from these data that there are approximately similar numbers of *Nicrophorus* beetles within each woodland competing for an approximately similar size, abundance, and species of rodent carrion to breed upon. The key difference lies in the number of burying beetle species in each wood, which is greater in Gamlingay Wood than in Waresley Wood. These data suggest that *N. vespilloides* in Gamlingay Wood is likely to be confined to breeding only on smaller carrion, whereas *N. vespilloides* from Waresley Wood should more routinely breed on larger carrion as well. We found no evidence from a mark‐recapture experiment that beetles moved between woodlands (see Supporting Information).

We tested this hypothesis using experiments in the laboratory to determine the carrion niche occupied by *Nicrophorus* species from the two woods. We analyzed the following species, in decreasing order of size (Fig. 1A): *N. investigator* (from Gamlingay Wood), *N. interruptus* (from Gamlingay Wood), and *N. vespilloides* beetles (from both Gamlingay and Waresley Woods) (see Methods). For each species, we presented breeding pairs with either a small mouse carcass (range: 12‐20 g; mean ± SEM: 16.80 ± 0.55 g) or a large mouse carcass (range: 25‐31 g; mean ± SEM: 28.27 ± 0.53 g; natural carcass range = 8.5‐41 g). To quantify reproductive performance on each carcass size, we measured “carcass use efficiency”, which we calculated by dividing the total brood mass at the end of larval development (measured when larvae had dispersed from the carcass) by the size of the carcass the brood was reared on. We predicted that *N. investigator* and *N. interruptus* should each exhibit greatest efficiency when breeding on a large carcass, whereas *N. vespilloides* from Gamlingay Wood should exhibit greatest efficiency when breeding on a small carcass. We further predicted that *N. vespilloides* from Waresley Wood should be more efficient at breeding on a large carcass than *N. vespilloides* from Gamlingay Wood.

**Figure 2:**
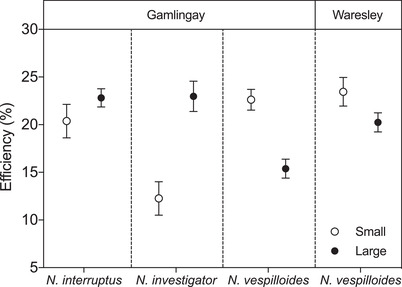
Efficiency (%) of carcass use (total brood mass divided by carcass mass) of *N. interruptus*, *N. investigator*, and *N. vespilloides* from Gamlingay Wood and *N. vespilloides* from Waresley Wood. Values represent the mean ± SEM.

We found that the efficiency of converting the carcass into larvae varied with carcass size, but in a different way for each *Nicrophorus* species (carcass size × *Nicrophorus* species interaction term: *χ*² = 28.85, d.f. = 3, *P* < 0.001; Fig. 2 and Table S3). *Nicrophorus investigator* exhibited greater efficiency when breeding on large carcasses rather than on small carcasses (*t* = 3.51, adj. *P* < 0.001), suggesting it is adapted to breed on larger carrion. *Nicrophorus interruptus* was similarly efficient when breeding on both large and small carcasses (*t* = 0.99, adj. *P* = 0.325), suggesting it is more of a generalist. In contrast, *N. vespilloides* from Gamlingay Wood exhibited greater reproductive efficiency on small carcasses (*t* = –4.16, adj. *P* < 0.001), and was less efficient at breeding on larger carcasses than both *N. interruptus* (*t* = –3.92, adj. *P* < 0.001) and *N. investigator* (*t* = –2.69, adj. *P* = 0.038). Therefore, we conclude that *N. vespilloides* from Gamlingay Wood is adapted to breed on smaller carrion.

In general, larger *N. vespilloides* beetles had greater reproductive efficiency (*χ*² = 6.09, d.f. = 1, *P* = 0.014). However, after controlling for body size, we found that Gamlingay *N. vespilloides* showed a greater loss in their reproductive efficiency than Waresley *N. vespilloides* when they were given larger carrion to breed upon (carcass size × woodland interaction term: *χ*² = 6.42, d.f. = 1, *P* = 0.011; Fig. 2). Although Waresley beetles were similarly efficient to Gamlingay beetles when breeding on a small carcass (*t* = 0.95, adj. *P* = 0.342), they were significantly more efficient at breeding on a large carcass than *N. vespilloides* from Gamlingay Wood (*t* = –2.65, adj. *P* = 0.009). We assume these differences are adaptive and conclude that the population of *N. vespilloides* in Waresley Wood occupies a broader carrion niche than *N. vespilloides* from Gamlingay Wood because it faces very little competition for larger carrion from *N. interruptus* and *N. investigator*.

### WARESLEY AND GAMLINGAY *N. vespilloides* HAVE DIVERGENT REACTION NORMS

**Figure 3:**
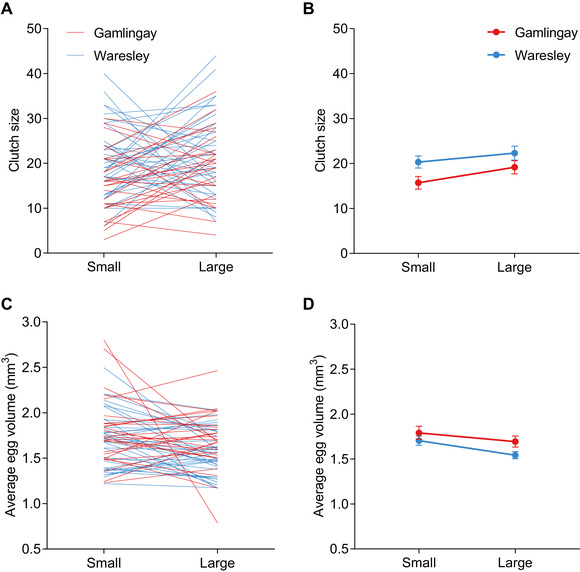
The effect of carcass size on (A and B) clutch size and (C and D) average egg volume produced by *N. vespilloides. n* = 27 Gamlingay *N. vespilloides* per carcass size treatment, and *n* = 37 Waresley *N. vespilloides* per carcass size treatment. (A) and (C) show reaction norms derived when siblings are exposed to Small and Large carcasses, with each line connecting siblings from the same brood; values in (B) and (D) represent the mean ± SEM of these reaction norms, so that population differences can more easily be seen.

To test whether the expansion of the carrion niche by Waresley *N. vespilloides* was facilitated by genetic accommodation, we generated reaction norms relating carcass size to clutch size for *N. vespilloides* from the two woodland populations. To derive reaction norms for each population, we compared the clutch sizes produced by sisters, when one sister was given a small carcass to breed upon and the other was given a larger carcass (see Methods and Fig. S4). From the results in Figure 2, we predicted that the reaction norm for Waresley *N. vespilloides* would be significantly steeper than the reaction norm for Gamlingay *N. vespilloides*. In fact, we found that the reaction norms differed in their elevation rather than in their slope (Figs. 3A and 3B). Although female *N. vespilloides* from both woodlands laid more eggs when given a larger carcass to breed upon (carcass size effect: *χ*² = 11.33, d.f. = 1, *P* = 0.001; Table S4), Waresley *N. vespilloides* consistently laid more eggs than Gamlingay *N. vespilloides* (woodland effect: *χ*² = 21.07, d.f. = 1, *P* < 0.001; Table S4).

Female *N. vespilloides* from both woodlands laid smaller eggs when given a larger carcass to breed upon (carcass size effect: *χ*² = 6.08, d.f. = 1, *P* = 0.014; Figs. 3C and 3D). Irrespective of carcass size (carcass size × woodland interaction: *χ*² = 0.25, d.f. = 1, *P* = 0.615), Waresley *N. vespilloides* tended to lay smaller eggs than Gamlingay *N. vespilloides*, although this difference was not statistically significant after controlling for female size (woodland effect: *χ*² = 3.11, d.f. = 1, *P* = 0.078).

In addition, *N. vespilloides* produced heavier larvae at dispersal when breeding on a large carcass than on a small carcass (*χ*² = 139.05, d.f. = 1, *P* < 0.001; Fig. S5; Table S4), again irrespective of their woodland of origin (*χ*² = 1.06, d.f. = 1, *P* = 0.304; Fig. S5; Table S4). This suggests that any under‐provisioning of eggs is compensated by the overabundance of resources available on the carcass after hatching (Russell et al. 2007; Smiseth et al. 2014). It further predicts that *N. vespilloides* adults from Waresley Woods should be larger than their counterparts in Gamlingay Woods, because they are more likely to have been raised on larger carrion. We found that this was indeed the case. *Nicrophorus vespilloides* trapped from Waresley Wood were significantly larger than *N. vespilloides* from Gamlingay Wood (*χ*² = 4.31, d.f. = 1, *P* = 0.038; Fig. S6A). We could not detect any differences in the variance in body size between Gamlingay and Waresley *N. vespilloides* (*D* = 0.039, *P* = 0.501; Fig. S6B), nor did the sexes differ in size (*χ*² = 0.96, d.f. = 1, *P* = 0.327).

### DIVERGENCE AT LOCI ASSOCIATED WITH OOGENESIS IN *N. vespilloides* FROM GAMLINGAY VERSUS WARESLEY WOODS

Next, we sought evidence of genetic divergence between Gamlingay and Waresley Woods that could be associated with differences between the woods in *N. vespilloides* egg production. We generated low‐coverage whole genome sequences for 40 diploid individuals collected from each wood. In general, we found a consistently low pattern of *F*_ST_ between populations (see Methods and Fig. S7), which suggests that there is little genetic differentiation between populations as a consequence of neutral processes such as drift. The highest *F*_ST_‐windows in the genome showed only modest absolute values of divergence, probably because the divergent traits are controlled by many loci. This is typical for behavioral and life history traits and consistent with the predicted quantitative genetic basis of genetic accommodation (Levis and Pfennig 2016). Nevertheless, there were notable outliers. For example, the top window of divergence between the two populations fell in *transmembrane protein 214* (*F*_ST_ = 0.11, *zF*_ST_ = 19.2, *P* = 7.2 × 10^–82^). This gene is highly expressed in the ovaries in *Drosophila melanogaster*, suggesting a potential candidate gene that may influence differences in *N. vespilloides* egg laying behavior between Gamlingay and Waresley Woods.

By comparing differentiation between both woodland populations and an outgroup population from 300 km away in Swansea, Wales, United Kingdom, we polarized the divergence between populations using population branch statistics (PBS). PBS calculates all pairwise *F*_ST_ values among the three populations and identifies differences in allele frequency relative to the other populations. Although elevated values of *F*_ST_ are indicative of a shift in allele frequency in one or both populations, elevated values of PBS allowed us to assign the relative divergence of each population to understand which population was driving divergence across the genome (Yi et al. 2010; Fumagalli et al. 2015). Not surprisingly, the distant Welsh population showed the highest genome‐wide PBS. We also found the population from Waresley Wood showed higher genome‐wide differentiation compared to the population from Gamlingay Wood (mean PBS: Waresley = 0.0074, Gamlingay = 0.0056, and Wales = 0.0109).

**Figure 4:**
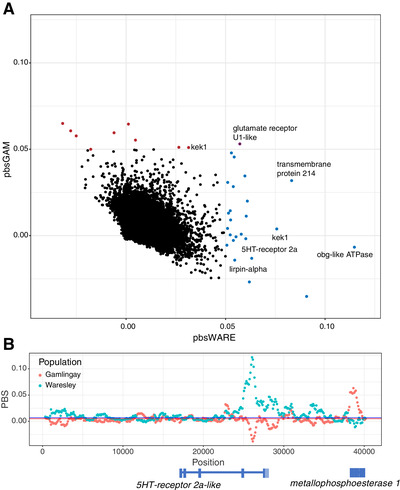
Differentiation at putative oogenesis genes. (A) Scatterplot of PBS values for Waresley and Gamlingay in 2‐kb windows genome‐wide. Loci in the lower right hand of the figure show high differentiation in Waresley but not in Gamlingay. Loci with PBS scores greater than 0.05 are highlighted—Waresley = blue, Gamlingay = red, and Both = purple. Notable genes are annotated. (B) Sliding window analysis (window = 500 bp; slide = 100 bp) of PBS values at the 5HT receptor 2a‐like receptor. The peak PBS in Waresley (blue) falls in the last intron of the gene.

To visualize the relative divergence between populations across the genome, we generated a scatterplot of the PBS values for 2‐kb nonoverlapping windows for each focal woodland population (Fig. 4). The analysis highlighted multiple potential candidate genes associated with the differences in egg‐laying behavior, again consistent with this trait being controlled by many loci of small effect. These genes showed elevated PBS in *N. vespilloides* from Waresley Wood (PBS > 0.05), but not Gamlingay Wood, and they are known to be linked to arthropod oogenesis. For example, homologs of three of the highly differentiated genes in the Waresley Wood population—*obg‐like ATPase*, *transmembrane protein 214*, and *liprin‐alpha*—show elevated expression in the ovaries of fruit flies (Chintapalli et al. 2013) and other arthropods (Cao and Jiang 2017), suggesting a plausible role in regulating egg production. Another gene, *kekkon1*, is a transmembrane protein known to regulate the activity of the epidermal growth factor receptor during oogenesis in *Drosophila* (Ghiglione et al. 1999; Wittes and Schüpbach 2019).

Finally, we asked whether genes involved in oogenesis generally showed elevated levels of differentiation in each population, in comparison with the rest of the genome. For each population, we ranked genes by the highest PBS score in 500‐bp windows overlapping with the gene body and conducted a gene set enrichment analysis for each population. *Nicrophorus vespilloides* from both Waresley and Gamlingay Woods showed enrichment in multiple GO terms associated with ovaries and oogenesis (Table 1). Because several of the divergent loci are related to oogenesis in other arthropods, and because there is little genetic differentiation in the rest of the genome, these analyses suggest that divergence between populations at these loci is adaptive. However, whether these genes are functionally related to egg‐laying behavior in *N. vespilloides* still remains to be determined in future work.

**Table 1:** Population branch statistic enrichment scores in multiple GO terms associated with ovaries and oogenesis in each population

			Waresley		Gamlingay		Wales	
GO ID	GO name	Size	NES	FDR *q*‐value	NES	FDR *q*‐value	NES	FDR *q*‐value
GO:0030707	Ovarian follicle cell development	345	3.36	0.00	3.27	1.2 × 10^–5^	2.52	1.35 × 10^–3^
GO:0007297	Ovarian follicle cell migration	129	3.32	0.00	2.86	1.0 × 10^–4^	3.09	4.10 × 10^–5^
GO:1905879	Regulation of oogenesis	66	2.22	7.31 × 10^–3^	2.07	1.4 × 10^–2^		
GO:0048599	Oocyte development	165	2.16	9.70 × 10^–3^	1.83	4.1 × 10^–2^		
GO:0007304	Chorion‐containing eggshell formation	78	2.12	1.20 × 10^–2^	2.41	2.0 × 10^–3^		
GO:0007308	Oocyte construction	157	2.07	1.55 × 10^–2^	1.83	4.2 × 10^–2^		
GO:0030703	Eggshell formation	79	2.04	1.80 × 10^–2^	2.50	1.3 × 10^–3^		
GO:1905881	Positive regulation of oogenesis	37	2.04	1.83 × 10^–2^				
GO:0009994	Oocyte differentiation	194	2.01	2.05 × 10^–2^	2.25	5.1 × 10^–3^		
GO:0007309	Oocyte axis specification	145	1.90	3.44 × 10^–2^	1.85	3.9 × 10^–2^		
GO:0030728	Ovulation	11	1.83	4.58 × 10^–2^				
GO:0007306	Eggshell chorion assembly	66			2.04	1.6 × 10^–2^		
GO:0060281	Regulation of oocyte development	29			1.79	4.9 × 10^–2^		

Notes: NES values report relative enrichment of PBS values for each GO term within each population. Higher NES values indicate that a gene set tends to be more near the top of the list. The values come from analyses within each population. The same gene sets are used in each population, although the relative distribution of population‐specific divergence across the genome varies among populations, leading to different scores. In comparison to the Welsh population, both Gamlingay and Waresley show many more GO terms related to oogenesis enriched at the top of their ranked gene list.

### SECONDARY LOSS OF SURPLUS OFFSPRING ON A SMALL CARCASS

**Figure 5:**
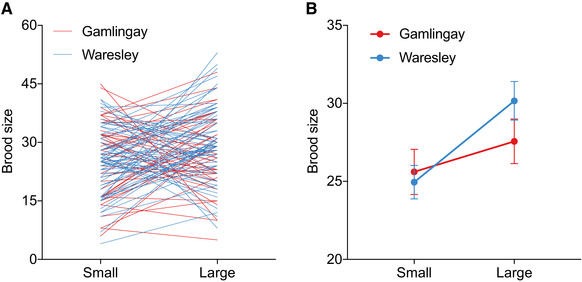
The effect of carcass size on the brood size produced by *N. vespilloides* (*n* = 46 Gamlingay *N. vespilloides* per carcass size treatment, and *n* = 62 Waresley *N. vespilloides* per carcass size treatment). (A) Reaction norms derived when siblings are exposed to Small and Large carcasses, with each line connecting siblings from the same brood; (B) Mean ± SEM of the values shown in (A), so that population differences can more easily be seen.

The change in clutch size does not fully account for the differences in reproductive efficiency that we found between Gamlingay and Waresley *N. vespilloides* (Fig. 2). It explains how Waresley *N. vespilloides* is able to increase its reproductive efficiency on a large carcass, but not how it also is able to breed efficiently on a small carcass. To address this problem, we analyzed further data from the reaction norm experiment. When we related brood size (measured at the end of reproduction) to carcass size, we found the predicted difference between Gamlingay and Waresley *N. vespilloides* in the slope of the reaction norm (woodland × carcass size interaction: *χ*² = 4.67, d.f. = 1, *P* = 0.031; Fig. 5; Table S4). On a large carcass, Waresley *N. vespilloides* produced more larvae than Gamlingay *N. vespilloides*, whereas on a small carcass the number of larvae produced did not differ between the two populations (Fig. 5). Therefore, although Waresley *N. vespilloides* lays more eggs on a small carcass than Gamlingay *N. vespilloides* (Fig. 3b), surplus offspring die after egg‐laying. Food is abundant on the carcass until larvae reach the third instar and resources become depleted. Nevertheless, larvae that are deprived of food at this developmental stage seldom starve to death (e.g., Steiger 2013). The death of excess larvae is therefore unlikely to be due to simple starvation. It is possible that some larval mortality could be due to bacterial or fungal infection (Arce et al. 2012), although we have no reason to think this would differ among populations, given the uniform breeding conditions in the laboratory. Instead, we think the differences in larval mortality are more likely to result from population differences in the extent of partial filial cannibalism (Bartlett 1987).

In short, Waresley *N. vespilloides* produces a larger clutch than Gamlingay *N. vespilloides* whatever the size of the carcass it locates for reproduction. This brings fitness gains if a Waresley beetle locates a large carcass because it can efficiently convert carrion into large numbers of offspring. Yet it brings fitness costs if a Waresley beetle instead finds a small carcass to breed upon, and then lays too many eggs. There are costs associated with the overproduction of offspring because there is a pronounced trade‐off between larval number and larval size when resources are limited (Schrader et al. 2015) and small larvae have markedly lower fitness (Lock et al. 2004). However, with a secondary mechanism for dispensing with excess young on smaller carcasses (probably filial cannibalism), these costs are eliminated. Therefore, the reaction norm relating clutch size to carrion is adaptive in Waresley Wood because it means *N. vespilloides* can breed efficiently on the larger carcasses that are available through the absence of *N. interruptus* and *N. investigator*. But it is only adaptive because there is an accompanying correcting mechanism that prevents the overproduction of offspring on a small carcass.

## Discussion

Our data suggest that the *N. vespilloides* populations in Gamlingay and Waresley Woods have divergently, locally, and recently adapted their clutch size to match their respective carrion niche, which in turn varies with the structure of the local burying beetle guild. Furthermore, our experimental results show that local adaptation is linked to divergence in the elevation of the reaction norm linking carrion size to clutch size. In addition, we have identified genetic differences between the two populations that may cause divergent expression of this trait.

To determine whether any local adaptation is due to “plasticity‐first” microevolution, we need to establish the direction of evolutionary change by identifying which of the two woodlands represents the ancestral proxy state, and which is the derived state (Levis and Pfennig 2016). Phylogeographic evidence, fossil evidence, and ecological evidence (set out in detail in the Supporting Information; Fig. S9) each strongly suggests that the burying beetle guild in the Wild Wood was richly speciose. Therefore, we consider the Gamlingay *N. vespilloides* population to be a proxy for the ancestral Wild Wood population, and that traits in the Waresley *N. vespilloides* population have more recently evolved from this baseline.

Assuming our inference is correct, we conclude that we have found evidence for three of the four criteria for “plasticity‐first” evolution (as set out in Levis and Pfennig 2016). Specifically, we have shown that clutch size is induced environmentally in the proxy ancestral population; that the elevation of this reaction norm has evolved; and that the derived population expresses a superior clutch size in a new, large‐carcass environment, in comparison with the ancestral population. We found no evidence for a fourth criterion, namely, that cryptic genetic variation in clutch size would be exposed (through greater phenotypic variance potentially) when Gamlingay *N. vespilloides* is induced to breed upon larger carcasses (see Fig. 3). The assumption behind this criterion is that genetic accommodation of the reaction norm proceeds through a change in its slope, which is dragged up or down by selection acting on previously unexpressed genetic variation in the new environment. However, this reasoning cannot explain an evolutionary increase in the elevation of a reaction norm because this involves expression of a new phenotype in both the “old” and the “new” environment. Evolutionary change in these circumstances is not solely due to selection acting on previously unexpressed genetic variation in a new environment.

Adaptive change, caused by plasticity‐first microevolution, appears to have enabled Waresley *N. vespilloides* beetles to expand their carrion niche in the absence of rival congeneric, in line with a central prediction of ecological character release theory (Grant 1972; Pfennig and Pfennig 2012). Although previous analyses of plasticity‐first evolution in wild animal populations have emphasized how new adaptations can evolve from the genetic assimilation of plastic traits (Scoville and Pfrender 2010; Levis and Pfennig 2016; Corl et al. 2018; Levis et al. 2018), our study is different in suggesting that adaptive traits in nature might also result from genetic accommodation. Furthermore, adaptation to the new environment in Waresley Wood has occurred despite evidence of recent gene flow with beetles from Gamlingay Wood (Pascoal and Kilner 2017). Although gene flow has traditionally been viewed as a force that disrupts adaptation, more recent analyses have shown that strong selection can maintain adaptive differences between populations even when they exhibit ongoing gene flow (Pinho and Hey 2010; Tigano and Friesen 2016; Pfeifer et al. 2018). Strong selection on clutch size could explain why we were able to find divergence in this trait between neighboring populations.

## Methods

### THE HISTORY OF GAMLINGAY AND WARESLEY WOODS

We focused on two woodlands: Gamlingay Wood (Latitude: 52.15555°; Longitude: −0.19286°) and Waresley Wood (Latitude: 52.17487°; Longitude: −0.17354°). They are woodland islands of approximately the same size (about 50 ha) in a landscape dominated by arable farming (Fig. S1A). In common with other woodlands recorded in the Domesday book, these woods have stayed approximately the same size since 1086 (Rackham 1996). Since then, Gamlingay Wood was acquired and managed by Merton College, Oxford for about 800 years (Adamson 1912). Its ecology was described in detail in 1912 (Adamson 1912). The modern history of Waresley Wood is less well‐known (Darby 1950; Rackham 1996). Both sites are now designated as “ancient woodland” and are managed by the Bedfordshire, Cambridgeshire, and Northamptonshire Wildlife Trusts.

### COMPETITION FOR CARRION IN GAMLINGAY AND WARESLEY WOODS

#### Burying beetle trapping

Carrion‐baited soil‐filled traps were suspended at each site, with traps set at least 150 m apart from each other. We collected and identified all the *Nicrophorus* spp. caught within each trap and measured the pronotum width (to the nearest 0.01 mm) as an index of body size (see below). All beetles were kept in the laboratory and were not re‐released to the field sites. We caught five species in total (in increasing order of size): *N. vespilloides*, *N. interruptus*, *N. vespillo*, *N. investigator*, and *N. humator*. We caught only five *N. vespillo* at four trapping events within the five years from 2014 to 2017 plus 2019, in Gamlingay and Waresley respectively, indicating that there is no stable population of *N. vespillo* in either wood. Ordination by NMDS (nonmetric multidimensional scaling) separated the guild structure between the woods for each sampling time across the five years (Fig. S1B). Evidence from other populations suggests that our measurements reflect long‐term differences in guild structure because abundance measures of *Nicrophorus* are robust over time (see Supporting Information).

#### Burying beetle husbandry in the lab

After removing any phoretic mites, beetles were retained and kept individually in plastic boxes (12 cm × 8 cm × 2 cm), which were filled with moist soil in a laboratory kept at 20°C and on a 16:8 light to dark cycle. Beetles were fed twice a week with minced beef. We kept all field‐caught individuals for at least two weeks before breeding to ensure that they were sexually mature and to reduce any variation in nutritional status. We then maintained stock populations of both Gamlingay and Waresley Woods by breeding pairs of unrelated individuals on 8‐16 g mice carcasses.

#### Size distributions of the *Nicrophorus* spp.

Body size was measured for *Nicrophorus* spp. collected from 2016, 2017, and 2019. In total, 908 *N. vespilloides*, 46 *N. humator*, 121 *N. interruptus*, 47 *N. investigator*, and five *N. vespillo* were measured for Gamlingay Wood, whereas 931 *N. vespilloides*, 48 *N. humator*, 32 *N. interruptus*, three *N. investigator*, and seven *N. vespillo* were measured for Waresley Wood. Mean body size of *Nicrophorus* spp. significantly varied among species (generalized linear mixed models [GLMM]: *χ*² = 1635.52, d.f. = 4, *P* < 0.001; Table S2). Post hoc comparisons revealed that *N. vespilloides* was smaller than the other *Nicrophorus* spp. (Tables S1 and S2). A Kolmogorov‐Smirnov test comparing pairwise differences in *Nicrophorus* spp. body size frequency distribution revealed similar patterns found in differences of mean body size (Fig. 1 and Table S5).

#### Small mammal trapping

To assess the rodent carrion available for *Nicrophorus* spp. reproduction, we sampled the small mammal communities in the two woodlands. In general, rodent populations peak in the autumn, because breeding for the year has just ceased and there has yet to be any winter‐induced mortality (Jackson et al. 2001). Sampling at this time is therefore ideal for detecting which species are present and for determining their relative abundance. We placed Longworth traps in both woodlands in November 2016. Traps were baited with oats and blowfly maggots (with hay provided as bedding) and set in pairs within 20 m of each original beetle trapping site (Fig. S1A), with 10 traps set per wood. We continuously trapped rodents for three days, generating 50 trap sessions per woodland. Traps were checked daily at approximately 0830h and 1500h (generating a total of 30 trap sessions overnight and 20 trap sessions in daylight hours). Trapped mammals were identified, weighed, sexed, marked by a fur clip on either the right or left rear flank, and released in situ. Any recaptured mammal was recorded in subsequent censuses. All traps were reset and rebaited immediately after checking. In addition to the results reported in the main text, one yellow‐necked mouse (*Apodemus flavicollis*; range: 14–45 g) and one common shrew (*Sorex araneus*; range: 5–14 g) were caught in Waresley. Based on previous work in the literature, we have no reason to think that the mortality of these rodent species should differ between woodlands that are in such close proximity and that are subjected to similar levels of ecological management (Hansson 1991; Harris and Yalden 2008).

#### Division of the carrion niche by *Nicrophorus* beetles in Gamlingay and Waresley Woods

The “carrion niche” occupied by burying beetles has been defined in previous work as the size range of carrion upon which burying beetles can successfully breed (Scott 1998). Absolute niche breadth can be characterized in laboratory experiments, through the experimental presentation of different carrion sizes. Smaller species are unable to breed on very large carrion, whereas larger species will not breed on small carrion (Trumbo 1990; Hopwood et al. 2016). In nature, the realized breadth of the carrion niche may be further constrained by competition with congenerics (Scott 1998).

During the field seasons in 2017 and 2018, pairs of wild‐caught *N. vespilloides*, *N. interruptus*, and *N. investigator* were bred on either small (12‐20 g; 16.80 ± 0.55 g) or large carcasses (25‐31 g; 28.27 ± 0.53 g) within a breeding box (17 cm × 12 cm × 6 cm) filled with 2 cm of moist soil. All field‐caught beetles were kept for two weeks in the laboratory and fed twice a week prior to breeding. In total, we established eight treatments: large (*n* = 47) and small (*n* = 48) carcasses for Gamlingay *N. vespilloides*; large (*n* = 42) and small (*n* = 33) carcasses for Waresley *N. vespilloides*; large (*n* = 25) and small (*n* = 18) carcasses for *N. interruptus*; large (*n* = 9) and small (*n* = 13) carcasses for *N. investigator*. *Nicrophorus interruptus* and *N. investigator* were both drawn from Gamlingay Wood as the populations of these species in Waresley were too small to be used experimentally.

Approximately eight days after parents are given a carcass to breed on, larvae switch from aggregating on the carcass to dispersing away into the soil to pupate. When one or more larvae from each brood switched their behavior in this way, we scored the whole brood as having reached the dispersal stage. At this point, all larvae were counted and total brood mass was weighed to the nearest 0.001 g. We also calculated average larval mass for each brood by dividing total brood mass by number of larvae. In addition, we calculated carcass use efficiency by dividing the total brood mass at dispersal by the size of the carcass the brood was reared on.

#### Deriving reaction norms that link carcass size to clutch size in *N. vespilloides*

This experiment was conducted in the laboratory, over two blocks in 2017, using the second and third descendant generations of field‐caught beetles from Gamlingay and Waresley Woods. By rearing beetles from both woodlands in the lab in a common garden environment for at least one generation prior to testing, we minimized any residual environmental effects when quantifying the reaction norm for each population.

To derive the reaction norms for each population, we compared the breeding performance of genetically standardized pairs on either a small or large carcass. We began by haphazardly casting unrelated broods into dyads, as soon as larvae were sexually mature adults at 2‐3 weeks after eclosion. From one brood in the dyad, we haphazardly chose two males; from the other brood, we haphazardly chose two females. The females were then paired with the males.

Two of these pairs were then given a small mouse to breed upon (12‐17 g; 15.03 ± 0.67 g), whereas the remaining two pairs were given a large mouse to breed upon (26‐31 g; 28.86 ± 0.67 g). By using sibships to generate pairs in this way, we were able to compare how very similar genotypes responded to the opportunity to breed on either a small or large mouse. Each pair, and their mouse, was housed in a clear plastic box (17 cm × 12 cm × 6 cm), with 2 cm depth of Miracle‐Gro compost. The box was placed in a dark cupboard for eight days after pairing the beetles, until larvae started to disperse away from the carcass. In the second block of the experiment, we measured clutch size. Fifty‐six hours after we introduced the beetles to the carcass, we photographed the base of each transparent breeding box. Using Digimizer version 5.1.0, we then counted the number of visible eggs, and also measured the length (*L*) and width (*w*) of all eggs that were able to be measured accurately (i.e., those that were fully visible and lying flat on the base of the box).

In total, 2518 eggs were counted across 132 breeding boxes, of which 1633 could be measured for size. No eggs could be seen in four of these boxes, and these were excluded from further analysis (hence *n* = 124). The remaining 124 clutches all yielded larvae. The total number of eggs counted (observed clutch size), and the number of eggs that could accurately be measured, each correlated positively with brood size (number of eggs counted: *χ*² = 27.18, d.f. = 1, *P* < 0.001; number of eggs measured: *χ*² = 24.26, d.f. = 1, *P* < 0.001), indicating these are accurate proxy measures of true clutch size.

Nevertheless, it is likely that we underestimated clutch size in some instances because some eggs were hidden in the soil and could not be seen from the underside of the box. To reduce error introduced in this way, we limited the volume of soil placed in each breeding box (2 cm depth, about 400 ml). We did not sift through the soil to count all eggs because this would disrupt the breeding process, risk egg destruction, and potentially destroy the crypt prepared by the parents. All counting and measuring of eggs were performed blind to the carcass size treatment and the population from whence the breeding beetles came. Egg volume was then calculated using the formula *V* = 1/6 × π × *w* ^2^ × *L*, which assumes eggs to be a prolate spheroid (following Berrigan 1991). In both blocks, we also measured the number of larvae present at dispersal and weighed the whole brood.

#### Divergence at loci associated with oogenesis in *N. vespilloides* from Gamlingay versus Waresley Woods

We generated low‐coverage whole genome sequences for three populations of *N. vespilloides*: from Waresley Wood, Gamlingay Wood and Swansea, Wales, United Kingdom. Twenty‐two *N. vespilloides* from three ancient woodlands near Swansea in Wales were trapped by Dr. Chris Cunningham in 2017. The three sites in Wales (Park Wood [Latitude: 51.57258°; Longitude: −4.03096°]; Clyne Valley Wood [Latitude: 51.61262°; Longitude: −4.02293°]; and Caswell Bay Wood [Latitude: 51.57258°; Longitude: −4.03096°]) are approximately 300 km away from our two study sites in Cambridgeshire.

DNA was individually extracted from beetle heads using the DNeasy Blood and Tissue kit (Qiagen) and subsequently quantified and quality checked using NanoDrop and Qubit. DNA was then shipped to Cornell University, where paired‐end 550 bp insert libraries were prepared using partial reactions of a Nextera kit by the Cornell Genomics Core. Libraries were subsequently sequenced by Novogene (Davis, CA, USA) at an average coverage of 3.4×. Trimmomatic (version 0.36) was used to removed adaptors and poor‐quality sequence. Trimmed reads were mapped to the *N. vespilloides* reference genome using the Burrows‐Wheeler Aligner (version 0.7.13) (Li and Durbin 2009). SNPs were identified using Picard (version 2.8.2) and GATK (version 3.6) HaplotypeCaller following best practice recommendations (Van der Auwera et al. 2013). After alignment, SNPs were hard filtered using the parameters: QualByDepth (QD) < 2.0 || StrandOddsRatio (SOR) > 3.0 || FisherStrand (FS) > 200. We used VCFtools to calculate population genetic statistics for each population. To examine population structure, we generated a thinned VCF file with one SNP per 5 kb and used Tassel (version 5) (Bradbury et al. 2007) to calculate generate an MDS plot (Fig. S8). For calculation of *F*_ST_ and PBS values, aligned bam files were analyzed in ANGSD (version 0.911) (Korneliussen et al. 2014), which is specifically designed for analysis of low‐coverage genome sequencing data. Data for this project are available at the NCBI Sequence Read Archive under Bioproject PRJNA530213.

Genes were assigned gene ontology (GO) terms using the BLAST2GO workflow (version 5.1.1). In brief, gene identity was determined based upon BLAST searches to the Arthropod or *Drosophila* nonredundant protein databases and protein domains were identified based on matches to Interpro database. GO terms were assigned to each gene model based upon mapping results. GO terms were filtered with the “Filter Annotation by GO Taxa” option to remove GO terms that are incompatible for Arthropods.

Population genetic summary statistics were similar for both populations (mean of 2 kb windows—Gamlingay nucleotide diversity (π) = 0.0055 ± 1.02 × 10^–5^, Tajima's D = –0.70 ± 0.002; Waresley π = 0.0056 ± 1.0 × 10^–5^, Tajima's D = –0.67 ± 0.002). Consistent with previous microsatellite analyses (Pascoal and Kilner 2017), we found little to no genetic differentiation between populations from Gamlingay and Waresley Woods (unweighted *F*_ST_ = 0.0069; weighted *F*_ST_ = 0.013), strongly suggesting there has been gene flow between the two populations.

To identify potential loci that differed between Gamlingay and Waresley populations, we used PBS analysis. To formally assess the extent to which there may or may not be an enrichment of divergence in loci with oogenesis‐annotated functions, we conducted a gene set enrichment analysis in Blast2GO for each population. This analysis does not use “cutoff” to consider gene enrichment, but rather uses a ranked list of genes based on some metric. In this case, each gene was assigned a divergence score based on the highest PBS value in a 500‐pb window that included the gene and then ranked from most to least diverged. PBS scores denote the population‐specific divergence, so each of the three populations can have a unique ranking of genes based on PBS values. Divergence in one population does not directly influence divergence in others, meaning that each population is a separate sample that we can analyze for gene set enrichment. Therefore, we examined gene set enrichment based on the PBS scores for all three populations separately and then compared the results of each analysis. All scores for each population are based on the enrichment of genes within a GO term at the top (or bottom for negative scores) of the ranked list of GO terms within a population. Thus, an enrichment analysis score does not compare PBS between populations, although relative patterns of enrichment scores across populations can indicate different patterns of evolution. For example, GO terms that are enriched in all populations suggest gene sets that are more rapidly diverging in general compared to the genome‐wide patterns. A GO term that is enriched in one population but the others would suggest population‐specific divergence. Populations can also vary in the relative enrichment of terms, with higher enrichment scores indicating more divergence in one population's gene set relative to its genome‐wide PBS scores compared to what is seen in the other populations. Consistent with the outlier analyses, oogenesis‐related GO terms tended to have higher enrichment scores in *N. vespilloides* from Waresley Wood compared to Gamlingay Wood (Table 1). The gene enrichment analysis also revealed local divergence between beetles from Waresley and Gamlingay Woods at genes associated with other traits, including learning and memory and sensory systems (Supporting Information Data 1).

### STATISTICAL ANALYSES

All statistical analyses were performed in R version 3.4.3 (R Development Core Team), with NMDS in the package *vegan*, generalized linear models (GLM) and GLMM in the package *lme4* (Bates et al. 2015), and Tukey's HSD post hoc comparisons in the package *lsmeans* (Lenth 2016).

#### Competition for carrion in Gamlingay and Waresley Woods: Field data

To test for a difference in the *Nicrophorus* guild between Gamlingay and Waresley Woods, a frequency table of beetle communities was analyzed using permutational multivariate analysis of variance (PERMANOVA) on two‐dimensional NMDS based on Bray‐Curtis distances (Clarke 2016), with the “adonis” function (vegan package). We tested the effect of study site on the composition of the beetle community, using sampling year as strata. The analysis was based on 10,000 permutations of the data. We visualized the difference of beetle community between Gamlingay and Waresley population in two dimensions of a NMDS plot. NMDS two‐dimensional stress values (a measure of goodness of fit) were below 0.1 (0.082), indicating the ordination provides a good fit to the data (Ramette 2007).

We used GLMM to test for differences across beetle species and sites on the number and relative abundance of each species per trap during the field seasons. Beetle species, population (Gamlingay/Waresley), sampling day, and their interactions were included as fixed effects, whereas trap ID and sampling year were included as random factors. For both analyses, we included the number of each species per trap as a response variable with a Poisson error structure. For the relative abundance, we additionally added an offset of the log total number of beetles caught per trap.

*Nicrophorus investigator* is considered a habitat generalist and so can potentially thrive outside woodlands. We were constrained in where we could set traps in Gamlingay Wood and, by chance, some were hung closer to the edge of the wood than any of those in Waresley Wood (see Fig. 1A and Supplementary Information). To test whether proximity to the woodland edge influenced the number of *N. investigator* trapped in Gamlingay Wood, we compared the number of *N. investigator* caught in the three traps nearer the edge of Gamlingay Wood (G2, G4, and G5) with those hung in more central locations (G1 and G3). We included the number of *N. investigator* per trap as the response variable with a Poisson error structure, trap location (closer to the woodland edge or not) and sampling day as fixed effects, and trap ID and sampling year as random factors. We further tested whether Gamlingay and Waresley Woods differed in the number of *N. investigator* caught for traps hung in central locations in each wood (i.e., G1 and G3 vs. W1‐W5). For this analysis, population (Gamlingay/Waresley) and sampling day were included as fixed effects, and trap ID and sampling year were included as random factors. The results are given in the Supporting Information.

We also tested for differences in the probability of co‐occurrence (co‐occurring = 1; non‐co‐occurring = 0) between *N. vespilloides* and at least one other *Nicrophorus* spp. across sites using a binomial GLMM. Population was included as a fixed effect and year as a random effect.

We compared the distributions of body size between Gamlingay and Waresley *N. vespilloides*, and among *Nicrophorus* spp., using the Kolmogorov‐Smirnov two‐sample test, which tests whether the cumulative distributions of two datasets are derived from the same distribution. We also tested for significant differences in mean body size between *N. vespilloides* in Gamlingay and Waresley, using a GLMM that included population (Gamlingay/Waresley) and sex (male/female) as fixed effects, and sampling year as a random factor. Differences of mean body size among *Nicrophorus* spp. was assessed in a GLMM that included species and sex as fixed effects, and sampling year as a random factor.

We analyzed the body mass of rodents in Gamlingay and Waresley using a GLM by including rodent species and population (Gamlingay/Waresley) as fixed effects.

#### Division of the carrion niche by *Nicrophorus* beetles in Gamlingay and Waresley Woods

To test for differences in reproductive performance between species, we conducted a GLMM regression to analyze differences in efficiency (total brood mass divided by carcass mass), which was logit transformed prior to analysis. Beetle species, carcass size (small/large), and their interaction were included as explanatory variables. Sampling year was included as a random factor. In this analysis, we included beetle species as *N. interruptus*, *N. investigator*, Gamlingay *N. vespilloides*, and Waresley *N. vespilloides* to fully compare differences not only between *Nicrophorus* beetle species, but also *N. vespilloides* between populations. A Tukey's post hoc test was performed to detect significant effects between carcass size and beetle species using multiple pairwise comparisons.

#### Comparing *N. vespilloides* reaction norms between Gamlingay and Waresley Woods

For the reaction norm experiment, we used GLMMs to test the interacting effect of population and carcass size on brood size and average larval mass, with dyad identity nested within block included as a random factor. Adult body size of female beetle was also included as a covariate. Beetles failed to produce larvae in 21 out of 237 breeding events (17 out of 109 from block one, and four out of 128 from block two), and these breeding failures were excluded from the analyses. In all models, brood size and average larval mass were analyzed with a Poisson and Gaussian error distribution, respectively. A similar statistical approach was used for analyses of clutch traits to test for the significant differences on clutch size and average egg volume in GLMMs with a Poisson and Gaussian error distribution, respectively. The effects of population of origin, carcass size, and their interaction were included as fixed effects, whereas dyad identity was included as a random factor. We also included adult body size as a covariate. If a significant interaction was found, a Tukey's post hoc test was performed to detect significant effects using multiple pairwise comparisons.

## AUTHOR CONTRIBUTIONS

RMK conceived the idea. S‐JS, AMC, SP, and RMK designed the study. S‐JS, AMC, and BJMJ acquired the phenotypic data, which was analyzed by S‐JS and AMC. MJS and SP designed the genetic component, which was collected by SP and analyzed by MJS and SEM. All authors contributed to discussion of the results and writing the manuscript.

## DATA ARCHIVING

All data can be found on the Dryad digital repository (https://doi.org/10.5061/dryad.msbcc2fvj).

## CONFLICT OF INTEREST

The authors declare no conflict of interest.

Associate Editor: A. Charmantier

## Supporting information


**Supplementary Fig. 1**. Study sites and population differences in community structure.
**Supplementary Fig. 2**. Heatmaps showing temporal and spatial population differences for (a) *N. vespilloides*, (b) *N. interruptus*, (c) *N. investigator* and (d) *N. humator*.
**Supplementary Fig. 3**. Mean body weight of small mammals
**Supplementary Fig. 4**. Experimental design for the reaction norm experiment.
**Supplementary Fig. 5**. The effect of carcass size on offspring size for *N. vespilloides* in the reaction norm experiment (*n* = 46 Gamlingay *N. vespilloides* per carcass size treatment, and *n* = 62 Waresley *N. vespilloides* per carcass size treatment).
**Supplementary Fig. 6**. Differences in (a) body size and (b) frequency distribution of body size in field‐caught *N. vespilloides* from Gamlingay and Waresley Woods.
**Supplementary Fig. 7**. Density plot of *F*_ST_ value between Gamlingay and Waresley Woods.
**Supplementary Fig. 8**. MDS plot of three burying beetle populations.
**Supplementary Table 1**. *Nicrophorus* spp. body size (given by pronotum size, in mm).
**Supplementary Table 2**. Post‐hoc Tukey HSD comparing mean body size between *Nicrophorus* spp.
**Supplementary Table 3**. Results of the ANOVAs for division of carrion niche by *Nicrophorus* spp.
**Supplementary Table 4**. Results of the ANOVAs for reaction norm experiment.
**Supplementary Table 5**. Differences in body size frequency distribution between *Nicrophorus* spp.
**Supplementary Data 1**. Spreadsheet file of gene set enrichment analysis results of the multiple GO terms for Gamlingay and Waresley *N. vespilloides*.
**Supplementary Table 6**. Fossil sites of *Nicrophorus* in Great Britain.
**Supplementary Fig. 9**. A map of fossil *Nicrophorus* sites in Great Britain; numbers correspond to the site numbering in Supplementary Table 6.

Supplementary Materia
