# Supplementary material for: Rapid local adaptation linked with phenotypic plasticity
Source: Evol Lett. 2020 May 27;4(4):345–59. doi: 10.1002/evl3.176 (PMC7403679; doi:10.1002/evl3.176)
Supplement: Supplementary file 1 — Supplementary Fig. 1. Study sites and population differences in community structure. Supplementary Fig. 2. Heatmaps showing temporal and spatial population differences for (a) N. vespilloides, (b) N. interruptus, (c) N. investigator and (d) N. humator. Supplementary Fig. 3. Mean body weight of small mammals Supplementary Fig. 4. Experimental design for the reaction norm experiment. Supplementary Fig. 5. The effect of carcass size on offspring size for N. vespilloides in the reaction norm experiment (n = 46 Gamlingay N. vespilloides per carcass size treatment, and n = 62 Waresley N. vespilloides per carcass size treatment). Supplementary Fig. 6. Differences in (a) body size and (b) frequency distribution of body size in field‐caught N. vespilloides from Gamlingay and Waresley Woods. Supplementary Fig. 7. Density plot of F ST value between Gamlingay and Waresley Woods. Supplementary Fig. 8. MDS plot of three burying beetle populations. Supplementary Table 1. Nicrophorus spp. body size (given by pronotum size, in mm). Supplementary Table 2. Post‐hoc Tukey HSD comparing mean body size between Nicrophorus spp. Supplementary Table 3. Results of the ANOVAs for division of carrion niche by Nicrophorus spp. Supplementary Table 4. Results of the ANOVAs for reaction norm experiment. Supplementary Table 5. Differences in body size frequency distribution between Nicrophorus spp. Supplementary Data 1. Spreadsheet file of gene set enrichment analysis results of the multiple GO terms for Gamlingay and Waresley N. vespilloides. Supplementary Table 6. Fossil sites of Nicrophorus in Great Britain. Supplementary Fig. 9. A map of fossil Nicrophorus sites in Great Britain; numbers correspond to the site numbering in Supplementary Table 6. [file EVL3-4-345-s001.docx]

**Supplementary Information**

**Supplementary Fig. 1. Study sites and population differences in community structure. a**, Location of traps in Gamlingay Wood (red points) and Waresley Wood (blue points). **b**, NMDS ordination of Gamlingay (*n* = 177) and Waresley (*n* = 174) community structure. Each point represents the sum of the beetle community collected in each trap per sampling time. The size of point is proportional to abundance of *Nicrophorus* spp. community.

**Supplementary Fig. 2. Heatmaps showing temporal and spatial population differences for (a) *N. vespilloides*, (b) *N. interruptus*, (c) *N. investigator* and (d) *N. humator*.** The trap IDs correspond to the five trapping locations in Gamlingay (G) and Waresley (W) woods, shown in Figure 1 in the main text. The colour intensity in each panel indicates the averaged number of individuals in each trap during each sampling period over the five-year study (see colour key for details).


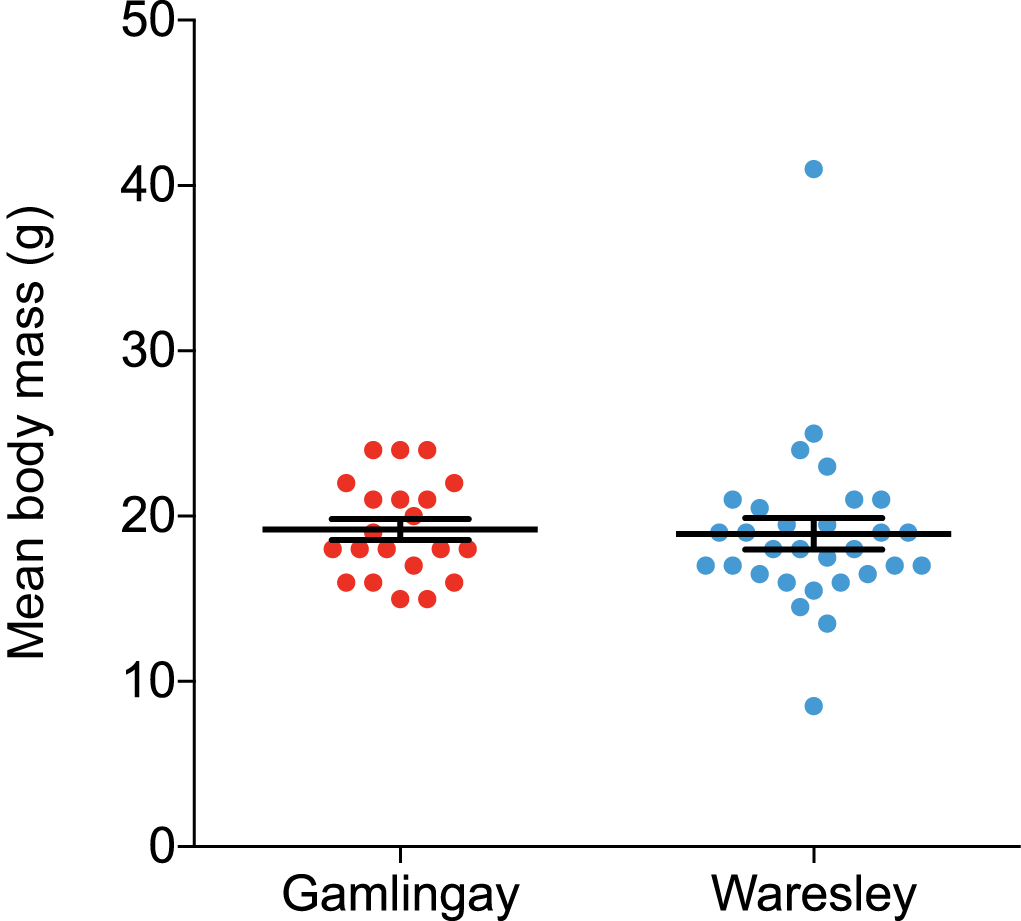


**Supplementary Fig. 3. Mean body weight of small mammals,** in Gamlingay (*n* = 21, red) and Waresley Woods (*n* = 30, blue). One bank vole and one wood mouse, each caught in Gamlingay Wood, escaped before they could be measured. The values represent the mean ± S.E.M, and each datapoint represents one trapping event.

**Supplementary Fig. 4.** **Experimental design for the reaction norm experiment.** Photo credit: Tom Houslay.

**Supplementary Fig. 5.** The effect of carcass size on offspring size for *N. vespilloides* in the reaction norm experiment (*n* = 46 Gamlingay *N. vespilloides* per carcass size treatment, and *n* = 62 Waresley *N. vespilloides* per carcass size treatment). The values represent the mean ± S.E.M.


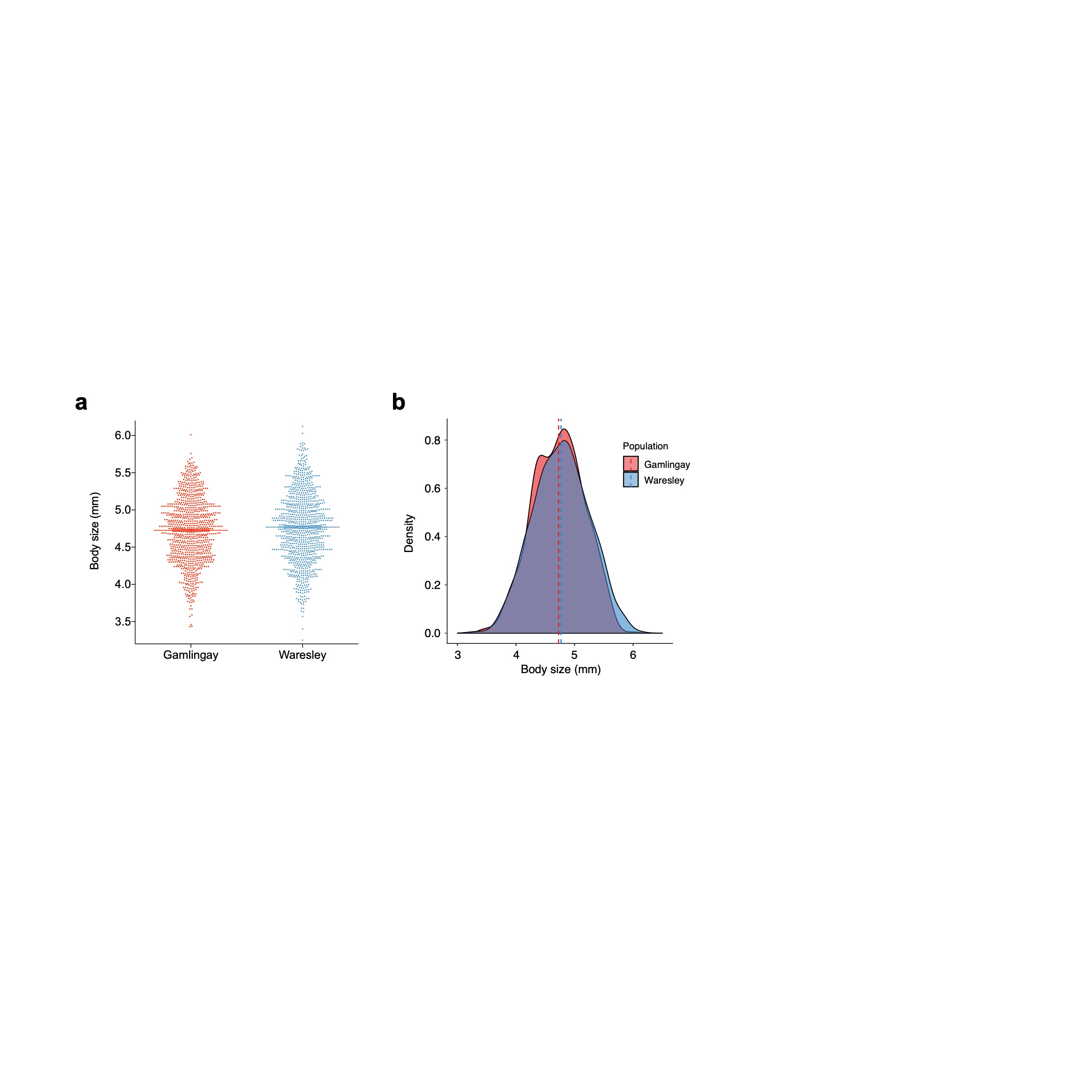


**Supplementary Fig. 6. Differences in (a) body size and (b) frequency distribution of body size in field-caught *N. vespilloides* from Gamlingay and Waresley Woods.** Measurements depict pronotum width, the standard index for measuring beetle body size. The values represent the mean ± S.E.M, and each datapoint represents one trapping event. Number of *N. vespilloides* sampled: Gamlingay (*n* = 908) and Waresley Woods (*n* = 931).

**
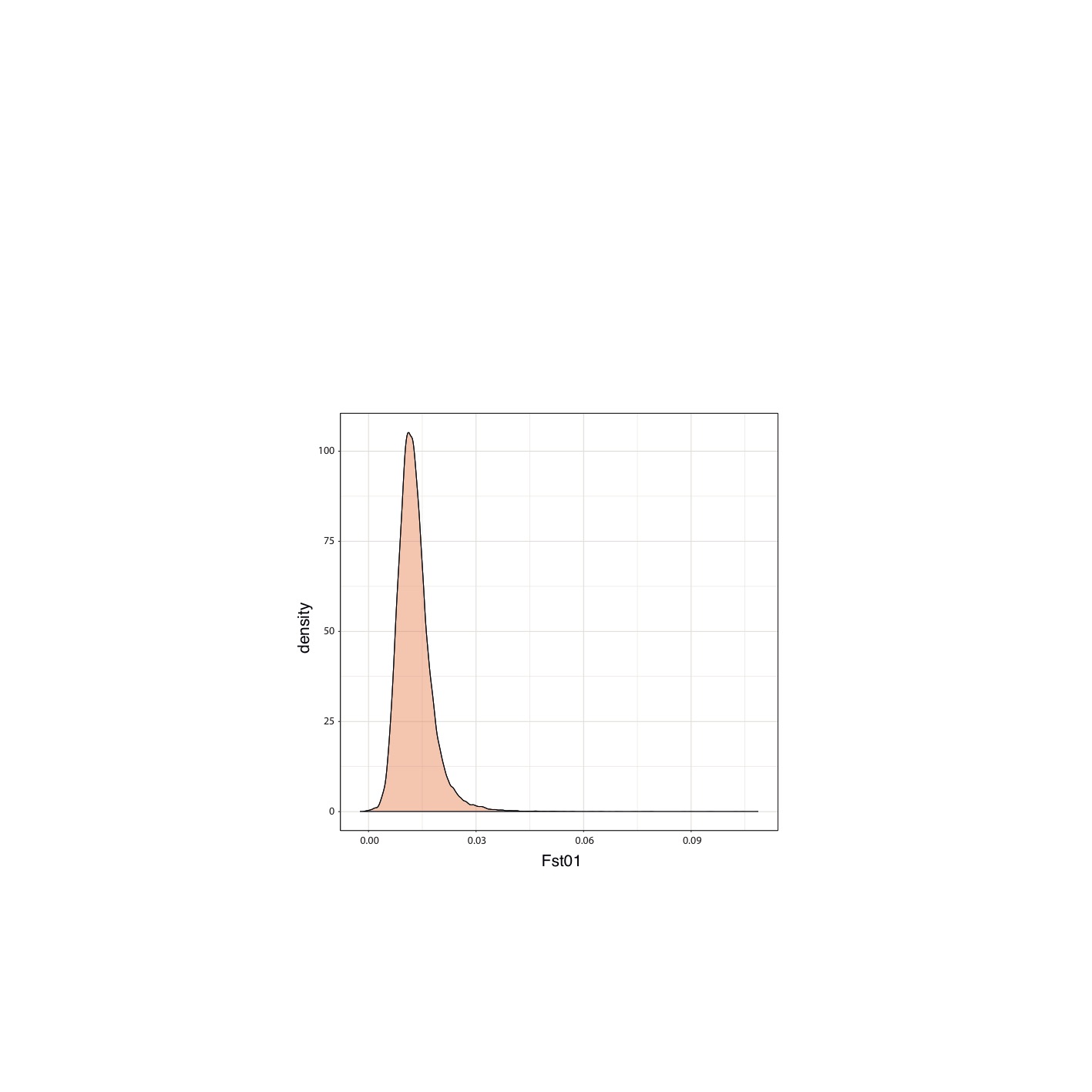
**

**Supplementary Fig. 7. Density plot of *Fst* value between Gamlingay and Waresley Woods.** The plot shows the narrow distribution of low *Fst* values between the two woodland populations. Though there is a long tail to the right, the extreme values are modest in absolute terms.

**Supplementary Fig. 8. MDS plot of three burying beetle populations.** The plot shows the first two dimensions of a multidimensional scaling analysis of genetic diversity among burying beetles in three populations. The plot indicates that the populations are statistically indistinguishable, indicating little to no genetic structure.

**Supplementary Table 1. *Nicrophorus* spp. body size (given by pronotum size, in mm).**

| **Species** | ***N*** | **Mean** | **SD** | **Minimum** | **Q1** | **Median** | **Q3** | **Maximum** |
| --- | --- | --- | --- | --- | --- | --- | --- | --- |
| *N. vespilloides* | 1839 | 4.75 | 0.46 | 3.25 | 4.42 | 4.76 | 5.07 | 6.12 |
| *N. interruptus* | 153 | 5.62 | 0.67 | 4.28 | 5.08 | 5.62 | 6.15 | 7.07 |
| *N. investigator* | 50 | 6.13 | 0.52 | 4.96 | 5.78 | 6.16 | 6.56 | 7.13 |
| *N. humator* | 94 | 6.93 | 0.84 | 5.20 | 6.32 | 6.91 | 7.39 | 9.39 |
| *N. vespillo* | 12 | 5.58 | 0.73 | 4.41 | 4.94 | 5.76 | 5.93 | 6.93 |

(*N*): Number of *Nicrophorus* spp.; (SD): Standard Deviation; 25% (Q1): Quantile; (Q3): 75% Quantile.

**Supplementary Table 2. Post-hoc Tukey HSD comparing mean body size between *Nicrophorus* spp.**

| **Species** | ***N. vespilloides*** | | ***N. interruptus*** | | ***N. investigator*** | | ***N. humator*** | |
| --- | --- | --- | --- | --- | --- | --- | --- | --- |
|  | *t* | *p* | *t* | *p* | *t* | *p* | *t* | *p* |
| *N. interruptus* | 20.52 | **<0.001** | - | - | - | - | - | - |
| *N. investigator* | 19.28 | **<0.001** | -6.31 | **<0.001** | - | - | - | - |
| *N. humator* | 41.19 | **<0.001** | 20.01 | **<0.001** | 9.14 | **<0.001** | - | - |
| *N. vespillo* | 5.69 | **<0.001** | 0.29 | 0.998 | 3.47 | **0.005** | 8.85 | **<0.001** |

*p* values <0.05 are highlighted in bold. Dashes indicate comparisons that were not made.

**Supplementary Table 3. Results of the ANOVAs for division of carrion niche by *Nicrophorus* spp.**

| **Dependent variable** | **Explanatory variables** | ***χ²*** | **d.f.** | ***p* value** |
| --- | --- | --- | --- | --- |
| Efficiency (%) | Carcass size | 17.26 | 1 | **<0.001** |
|  | Beetle species | 20.42 | 3 | **<0.001** |
|  | Carcass size x Beetle species | 28.85 | 3 | **<0.001** |

*p* values <0.05 are highlighted in bold.

**Supplementary Table 4. Results of the ANOVAs for reaction norm experiment.**

| **Dependent variable** | **Explanatory variables** | ***χ²*** | **d.f.** | ***p* value** |
| --- | --- | --- | --- | --- |
| Brood size | Carcass size | 3.36 | 1 | 0.067 |
|  | Population | 3.55 | 1 | 0.060 |
|  | Body size | 0.10 | 1 | 0.756 |
|  | Carcass size x Population | 4.67 | 1 | **0.031** |
| Average larval mass | Carcass size | 139.20 | 1 | **<0.001** |
|  | Population | 0.80 | 1 | 0.370 |
|  | Body size | 0.66 | 1 | 0.417 |
| Clutch size | Carcass size | 11.33 | 1 | **<0.001** |
|  | Population | 21.07 | 1 | **<0.001** |
|  | Body size | 4.26 | 1 | **0.039** |
| Average egg volume | Carcass size | 6.08 | 1 | **0.014** |
|  | Population | 3.11 | 1 | 0.078 |
|  | Body size | 10.46 | 1 | **0.001** |
| Total egg volume | Carcass size | 2.56 | 1 | 0.110 |
|  | Population | 3.32 | 1 | 0.068 |
|  | Body size | 6.27 | 1 | **0.012** |

*p* values <0.05 are highlighted in bold.

**Supplementary Table 5. Differences in body size frequency distribution between *Nicrophorus* spp.**

| **Species** | ***N. vespilloides*** | | ***N. interruptus*** | | ***N. investigator*** | | ***N. humator*** | |
| --- | --- | --- | --- | --- | --- | --- | --- | --- |
|  | *D* | *p* | *D* | *p* | *D* | *p* | *D* | *p* |
| *N. interruptus* | 0.53 | **<0.001** | - | - | - | - | - | - |
| *N. investigator* | 0.85 | **<0.001** | 0.36 | **<0.001** | - | - | - | - |
| *N. humator* | 0.94 | **<0.001** | 0.62 | **<0.001** | 0.47 | **<0.001** | - | - |
| *N. vespillo* | 0.57 | **<0.001** | 0.17 | 0.922 | 0.41 | 0.077 | 0.71 | **<0.001** |

D statistic and corresponding *p*-values from Kolmogorov-Smirnov tests. *p* values <0.05 are highlighted in bold. Dashes indicate comparisons that were not made.

**Supplementary Data 1. Spreadsheet file of gene set enrichment analysis results** **of the multiple GO terms for Gamlingay and Waresley *N. vespilloides*.**

**Supplementary Results**

**Mark-recapture experiment**

In 2014, we investigated the interconnectivity of populations of burying beetles between Gamlingay and Waresley Woods using a mark-recapture survey. In total, 98 *N. vespilloides*, 9 *N. humator*, 17 *N. interruptus*, 9 *N. investigator,* and 2 *N. vespillo* were marked for Gamlingay Wood, whereas 113 *N. vespilloides*, 5 *N. humator*, 1 *N. interruptus*, 1 *N. investigator* were marked for Waresley Wood. Beetles were marked with a numbered plastic bee tag on either the right or left elytra for those found in Gamlingay or Waresley Woods respectively. To identify any previously caught beetles that lost their tags, we also permanently marked them by cutting a small portion of the elytra (less than 2%). All marked beetles from each wood were released from a designated location at the geographic midpoint with the minimum total distance to all trapping sites for Gamlingay (Latitude: 52.16294°; Longitude: −0.18984°) and Waresley Woods (Latitude: 52.176508°; Longitude: −0.156776°). We found no evidence of migration between the two sites. We recaught 8 of 98 marked *N. vespilloides* from Gamlingay Wood in Gamlingay Wood and 8 out of 113 marked *N. vespilloides* from Waresley Wood in Waresley Wood. None of the other marked *Nicrophorus* spp. was recaptured in either wood.

**The *Nicrophorus* guild differs between Gamlingay and Waresley Woods**

Gamlingay and Waresley woodlands are both publically used nature reserves managed by Bedfordshire, Cambridgeshire, and Northamptonshire Wildlife Trusts, meaning that we had limited freedom in choosing where to locate traps and the number we could set. We distributed traps within each site to minimize the chance they would be discovered and tampered with by setting them at least 150 m apart. This resulted in some traps in Gamlingay Woods being set slightly closer to the woodland edge, which could potentially influence our trapping results for *N. investigator* which is considered to be a habitat generalist. However, we found no evidence that *N. investigator* was more likely to be caught at the edge of Gamlingay Wood than in more central locations (*χ²* = 0.14, d.f. = 1, *p* = 0.712). Furthermore, focusing on the traps positioned closer to woodland centre in Gamlingay (G1 and G3), we found that they trapped more *N. investigator* than all the traps in Waresley (*χ²* = 18.84, d.f. = 1, *p* < 0.001). These results suggest that even though some of our traps were placed closer to woodland edges for logistical reasons, trap location did not bias the differences we detected in the burying beetle guild in Gamlingay and Waresley Woods.

**Niche expansion in *N. vespilloides* by genetic accommodation**

***Clutch size***

Larger beetles produced a greater total volume eggs (Supplementary Table 4). There was also a tendency for Waresley females to produce a greater total volume of eggs than Gamlingay females (Supplementary Table 4). There was no difference in total volume of eggs between carcass size treatments (Supplementary Table 4).

***Brood size***

We found that when given a larger carcass for reproduction, *N. vespilloides* from Gamlingay Wood tended to produce larger brood than when breeding on a small carcass, but the effect was statistically non-significant (*z* = 1.82, *p* = 0.068). By contrast, Waresley *N. vespilloides* produced significantly larger broods on larger carcasses than on smaller carcasses (*z* = 5.53, *p* < 0.001). Whether they bred on small or large carcasses, the variance was similar for Gamlingay and Waresley *N. vespilloides* in both brood size (Bartlett’s test, *p* = 0.615) and carcass use efficiency (Bartlett’s test, *p* = 0.943)*.*

**Reconstructing the ancestral guild of *Nicrophorus* beetles in British woodlands**

A key challenge in testing ‘plasticity-first’ evolution is to distinguish between the ancestral population, or a proxy population for the ancestral state, and the derived population or state. The evidence below suggests that the *Nicrophorus* guild in Gamlingay Woods represents a proxy for the ancestral state, where the *Nicrophorus* guild in Waresley Woods represents the derived state.

1. **Phylogeographic evidence**

**• The species analysed in this study have co-existed in the Old World since their origin**

Molecular phylogeographic data support an Old World origin for *N. humator*, *N. interruptus*, *N. investigator* and *N. vespillo* [1]. The phylogeographic history of the *vespilloides* clade is more complicated following the taxonomic resurrection of *N.* *hebes*, but the forest-dwelling lineage (*vespilloides* sensu stricto; the lineage present in Britain) is thought to be Old World in origin [2]. There is no evidence for any very recent colonisations by these species from the New to the Old World.

1. **Fossil evidence**

**• Four of the five British burying beetles in this study are found in the British fossil record**

There are at least 20 fossil^[[1]](#footnote-1)^ *Nicrophorus* sites in Britain, which are widely distributed in time and space (see Supplementary Fig. 8. and Supplementary Table 6.). Of the five species of *Nicrophorus* in our study (*humator*, *interruptus*, *investigator*, *vespilloides*, and *vespillo*) only *investigator* has not been found as a fossil. This suggests that multiple *Nicrophorus* species have co-existed in Britain for centuries and that the Wild Wood had a speciose burying beetle guild. The predominance of larger-bodied burying beetles fossil remains may be due to the negative correlation between size and the rate of disarticulation (which will determine the likelihood of fossils of a species being preserved) which has been experimentally demonstrated in beetles [3].

The absence of a positively identified *N. interruptus* specimen may be due to general difficulties in identifying this species. For example, only 177 current records exist nationally [4] (c.f. 2,607 for *humator*, 2,502 for *vespilloides*, 1,475 for *investigator*, 876 for *vespillo*). Furthermore, seven of the 20 sites include unidentified *Nicrophorus* specimens.

**Supplementary Table 6.** Fossil sites of *Nicrophorus* in Great Britain. Full references for each site are found in the BugsCEP database [5].

| Site | Date | *Nicrophorus* taxa present | References |
| --- | --- | --- | --- |
| 1. Stansted, Essex | Bronze Age (c. 2500 BC – c. 800 BC) | *humator* x 1 | [6] |
| 1. Whitton, Radnorshire | 350 AD | *humator* x 1 | [7] |
| 1. Rearsby, Leicestershire | Roman Britannia (43 AD – 410 AD) | *humator* x1 | [5] |
| 1. Caerwent, Monmouthshire | Roman Britannia (43 AD – 410 AD) | *humator* x 1 | [8], [9] |
| 1. Edinburgh, Midlothian | Medieval (400 AD - 1550 AD) | *humator* x 2 | [10] |
| 1. London, Middlesex | Saxo-Norman (1060 AD – 1100 AD) | *humator* x 1 | [11] |
| 1. Brandon, Warwickshire | Marine Isotope Stage 6 (191 ka – 130 ka) | *investigator* x 1 | [12], [13] |
| 1. Bearsden, Dunbartonshire | 142 – 158 AD | *investigator* x 1 | [14] |
| 1. Afon Wen, Caernarfonshire | 12.625 ± 0.230 ka | *vestigator* x 2 | [15] |
| 1. Chelford, Cheshire | 60.8 ± 1.5 ka | *vespilloides* x 1 | [16], [17] |
| 1. Hoxne, Suffolk | Marine Isotope Stage 11 (424,000 ka - 374,000 ka) | *vespillo* x 1 | [18] |
| 1. Isleworth, Middlesex | 43.14 ± 1.52 ka | *vespillo* x 2 | [19] |
| 1. Wilsford, Wiltshire | 3.399 ± 0.09 ka | *vespillo* x 2 | [20] |
| 1. Pode Hole, Northamptonshire | 3.259 ± 0.04 ka | *vespillo* x 1 | [5] |
| 1. St Bees, Cumbria | Lateglacial (c. 13.5 ka – c. 10 ka) | *humator* x 1  *vespilloides* x 1  sp indet x ? | [21] |
| 1. Westward Ho!, Devon | Mesolithic and onwards (15 ka onwards) | spp. indet. x ? | [22] |
| 1. Munford, Norfolk | Marine Isotope Stage 3 (57 ka – 29 ka) | sp. indet. x 1 | [23] |
| 1. Cwmystwyth, Cardiganshire | 2.919 ± 0.08 ka | sp. indet. x 1 | [24] |
| 1. Preston-under-Scar, Yorkshire | 2.119 ± 0.1 ka | sp. indet^[[2]](#footnote-2)^. x 1 | [25] |
| 1. Droitwich Spa, Worcestershire | 270 AD | sp. indet. x 1 | [26] |
|  | 360 AD | sp. indet. x 1 |  |

**Supplementary Fig. 9.** A map of fossil *Nicrophorus* sites in Great Britain; numbers correspond to the site numbering in Supplementary Table 6.

1. **Ecological evidence**

**• Large, pristine forests support more speciose beetle communities**

The Wild Wood grew up following the retreat of the last glaciers from Britain c.13 kya, and comprised a series of woodland climax-communities that continuously covered most of the country. Given its vast size, the species-area relationship [27] predicts that the burying beetle guild in the Wild Wood would have been highly speciose. This relationship is known to hold for current populations of carrion beetles, including guilds of burying beetles (*Nicrophorus* spp.). A survey at 24 locations in the USA found that habitat size was positively correlated with species richness, and explained 83% of variation in this measure [28]. Furthermore, carrion beetle species richness was found to be greater in contiguous forests than in forest fragments [28]. Experimentally-induced habitat fragmentation had a disproportionately negative impact on larger forest-dwelling beetle species [29].

Deforestation of the Wild Wood began with the arrival of agriculturalists from the Eurasian steppe [30] c.4 – 5 kya. By the early Iron Age (c. 2.5 kya), the Wild Wood had probably halved in size [31]. As the Wild Wood was being destroyed during this period, about forty species^[[3]](#footnote-3)^ of beetle in Great Britain went extinct [32].

**References**

[1] D. S. Sikes and C. Venables, “Molecular phylogeny of the burying beetles (Coleoptera: Silphidae: Nicrophorinae),” *Molecular Phylogenetics and Evolution*, vol. 69, no. 3, pp. 552–565, Dec. 2013.

[2] D. Sikes, S. Trumbo, and S. Peck, “Cryptic diversity in the New World burying beetle fauna: Nicrophorus hebes Kirby; new status as a resurrected name (Coleoptera: Silphidae: Nicrophorinae),” *Arthropod Systematics and Phylogeny*, vol. 4, pp. 299–309, Dec. 2016.

[3] D. M. Smith, A. Cook, and C. R. Nufio, “How Physical Characteristics of Beetles Affect their Fossil Preservation,” *PALAIOS*, vol. 21, no. 3, pp. 305–310, Jun. 2006.

[4] “National Biodiversity Network Atlas,” *NBN Atlas*, 2019. [Online]. Available: https://nbnatlas.org/. [Accessed: 19-Sep-2019].

[5] P. I. Buckland and P. C. Buckland, “Bugs Coleopteran Ecology Package (Versions: BugsCEP v7.63; Bugsdata v9.4; BugsMCR v2.02; BugStats v1.22) [DOWNLOADED: September 2019],” 2006. [Online]. Available: https://www.bugscep.com/.

[6] M. Robinson, “Insect remains,” in *From Hunter Gatherers to Huntsmen: a history of the Stansted landscape*, Framework Archaeology, 2008.

[7] P. J. Osborne, “The insect fauna,” in *Whitton: an Iron Age and Roman farmstead in south Glamorgan*, University of Wales Press Cardiff, 1981.

[8] A. F. Amsden and G. C. Boon, “CO Waterhouse’s list of insects from Silchester: With a note on early identifications of insects in Archaeological contexts,” *Journal of Archaeological Science*, vol. 2, no. 2, pp. 129–136, 1975.

[9] T. Ashby, A. E. Hudd, and F. King, “XIX.— Excavations at Caerwent, Monmouthshire, on the site of the Romano-British City of Venta Silurum, in the years 1909 and 1910.,” *Archaeologia*, vol. 62, no. 2, pp. 405–448, ed 1911.

[10] E. Jones, “Through the Cowgate: life in 15th-century Edinburgh as revealed by excavations at St Patrick’s Church,” *Society of Antiquaries of Scotland*, vol. 42, 2010.

[11] D. N. Smith, *Insects in the City: An Archaeoentomological Perspective on London’s Past*. Archaeopress, 2012.

[12] G. R. Coope and F. W. Shotton, “An insect fauna from Mid-Weichselian deposits at Brandon, Warwickshire,” *Philosophical Transactions of the Royal Society of London. Series B, Biological Sciences*, vol. 254, no. 796, pp. 425–456, Dec. 1968.

[13] D. Maddy, G. R. Coope, P. L. Gibbard, C. P. Green, and S. G. L. Group, “Reappraisal of Middle Pleistocene fluvial deposits near Brandon, Warwickshire and their significance for the Wolston glacial sequence,” *Journal of the Geological Society*, vol. 151, no. 2, pp. 221–233, Mar. 1994.

[14] D. J. Breeze, *Bearsden: a Roman fort on the Antonine Wall*. Society of Antiquaries of Scotland, National Museum of Scotland, 2016.

[15] G. R. Coope and J. A. Brophy, “Late Glacial environmental changes indicated by a coleopteran succession from North Wales,” *Boreas*, vol. 1, no. 2, pp. 97–142, 1972.

[16] G. R. Coope, “A late Pleistocene insect fauna from Chelford, Cheshire,” *Proceedings of the Royal Society of London. Series B. Biological Sciences*, vol. 151, no. 942, pp. 70–86, 1959.

[17] G. R. Coope, “Fossil coleopteran assemblages as sensitive indicators of climatic changes during the Devensian (Last) cold stage,” *Philosophical Transactions of the Royal Society of London. B, Biological Sciences*, vol. 280, no. 972, pp. 313–340, 1977.

[18] R. Singer, B. G. Gladfelter, and J. Wymer, *The Lower Paleolithic Site at Hoxne, England*. University of Chicago Press, 1993.

[19] G. R. Coope and R. B. Angus, “An Ecological Study of a Temperate Interlude in the Middle of the Last Glaciation, Based on Fossil Coleoptera from Isleworth, Middlesex,” *Journal of Animal Ecology*, vol. 44, no. 2, pp. 365–391, 1975.

[20] P. J. Osborne, “An Insect Fauna of Late Bronze Age Date from Wilsford, Wiltshire,” *Journal of Animal Ecology*, vol. 38, no. 3, pp. 555–566, 1969.

[21] R. G. Pearson, “The Coleoptera from a Late-Glacial Deposit at St. Bees, West Cumberland,” *Journal of Animal Ecology*, vol. 31, no. 1, pp. 129–150, 1962.

[22] M. Robinson and M. Girling, “The Insect Fauna,” in *Prehistoric and Romano-British sites at Westward Ho!, Devon: archaeological and palaeoenvironmental surveys 1983 and 1984*, 1987, pp. 163–264.

[23] W. A. Boismier, C. Gamble, and F. Coward, *Neanderthals among mammoths: excavations at Lynford Quarry, Norfolk UK*. English Heritage Monographs, 2012.

[24] T. M. Mighall, S. Timberlake, S. H. E. Clark, and A. E. Caseldine, “A Palaeoenvironmental Investigation of Sediments from the Prehistoric Mine of Copa Hill, Cwmystwyth, mid-Wales,” *Journal of Archaeological Science*, vol. 29, no. 10, pp. 1161–1188, Oct. 2002.

[25] D. Jaques, “Technical report: biological remains from a site at Bolton Hall, Bolton, East Riding of Yorkshire (site code: TSEP238),” *Reports from the Environmental Archaeology Unit, York*, vol. 4, no. 2002, p. 31, 2002.

[26] J. Hughes, “Roman Droitwich: Dodderhill Fort, Bays Meadow Villa, and Roadside Settlement,” Council for British Archeology, York, 146, 2006.

[27] M. V. Lomolino, “Ecology’s most general, yet protean 1 pattern: the species-area relationship,” *Journal of Biogeography*, pp. 17–26, 2019.

[28] J. P. Gibbs and E. J. Stanton, “Habitat Fragmentation and Arthropod Community Change: Carrion Beetles, Phoretic Mites, and Flies,” *Ecological Applications*, vol. 11, no. 1, pp. 79–85, 2001.

[29] K. F. Davies, C. R. Margules, and J. F. Lawrence, “Which Traits of Species Predict Population Declines in Experimental Forest Fragments?,” *Ecology*, vol. 81, no. 5, pp. 1450–1461, 2000.

[30] I. Olalde *et al.*, “The Beaker phenomenon and the genomic transformation of northwest Europe,” *Nature*, vol. 555, no. 7695, pp. 190–196, Mar. 2018.

[31] O. Rackham, *The history of the countryside*. Dent London, 1986.

[32] N. Whitehouse, “The Holocene British and Irish ancient forest fossil beetle fauna: implications for forest history, biodiversity and faunal colonisation,” *Quaternary Science Reviews*, vol. 25, pp. 1755–1789, Aug. 2006.

1. These particular remains are more accurately sub-fossils, being primarily disarticulated exoskeletal remains. [↑](#footnote-ref-1)
2. “?vespillo” [25] [↑](#footnote-ref-2)
3. Of which over 60% are from taxa associated with old and dead wood (i.e. ‘wildwood’ taxa), with the rest representing other habitat types e.g. wetlands/meadow[33]. [↑](#footnote-ref-3)
